# Machine learning detection of obstructive hypertrophic cardiomyopathy using a wearable biosensor

**DOI:** 10.1038/s41746-019-0130-0

**Published:** 2019-06-24

**Authors:** Eric M. Green, Reinier van Mourik, Charles Wolfus, Stephen B. Heitner, Onur Dur, Marc J. Semigran

**Affiliations:** 1 MyoKardia, Inc., South San Francisco, CA USA; 2 Wavelet Health, Mountain View, CA USA; 3 Oregon Health Sciences University, Portland, OR USA

**Keywords:** Diagnostic markers, Translational research

## Abstract

Hypertrophic cardiomyopathy (HCM) is a heritable disease of heart muscle that increases the risk for heart failure, stroke, and sudden death, even in asymptomatic patients. With only 10–20% of affected people currently diagnosed, there is an unmet need for an effective screening tool outside of the clinical setting. Photoplethysmography uses a noninvasive optical sensor incorporated in commercial smart watches to detect blood volume changes at the skin surface. In this study, we obtained photoplethysmography recordings and echocardiograms from 19 HCM patients with left ventricular outflow tract obstruction (oHCM) and a control cohort of 64 healthy volunteers. Automated analysis showed a significant difference in oHCM patients for 38/42 morphometric pulse wave features, including measures of systolic ejection time, rate of rise during systole, and respiratory variation. We developed a machine learning classifier that achieved a C-statistic for oHCM detection of 0.99 (95% CI: 0.99–1.0). With further development, this approach could provide a noninvasive and widely available screening tool for obstructive HCM.

## Introduction

Hypertrophic cardiomyopathy (HCM) is a heritable disease of heart muscle characterized by hypertrophy without a systemic etiology. Patients with HCM are at increased risk of heart failure, stroke and sudden cardiac death.^1^ Approximately 30–40% of HCM patients have outflow tract obstruction (oHCM) at rest,^2^ which is associated with worse clinical outcomes.^3^ Characteristic hemodynamic abnormalities in patients with oHCM were noted in the earliest descriptions of HCM^4^ and have been observed using arterial pressure tracings, acoustic phonography, and echocardiography.

Echocardiographic screening, the standard diagnostic for HCM, estimates the prevalence of HCM at ~1:500 individuals.^5^ However, Medicare claims data identify only ~100,000 US patients diagnosed with HCM—an implied diagnosis rate of ~16%.^6^ Consistent with these data, in a study of sudden death victims diagnosed with HCM at autopsy, only ~20% had been clinically recognized.^7^ Efforts to use electrocardiography (ECG) or echocardiography to screen asymptomatic individuals for HCM have been limited by test characteristics and cost.^8,9^ Further, the nonspecific symptoms of HCM (exercise intolerance, dyspnea, and fatigue) can delay referral of even symptomatic individuals for diagnostic cardiac imaging studies. As a result, there is a demonstrable need for approaches to properly direct potential unrecognized patients for definitive diagnostic studies.

Photoplethysmography (PPG) is a noninvasive optical method to detect blood volume changes in the microvascular bed at the skin surface.^10^ This technology is the basis for clinical pulse oximeters and has now been widely incorporated into widely used commercial smartwatches for heart rate detection. We hypothesized that computational analysis of PPG waveforms collected from a wearable biosensor could distinguish between traces from patients with oHCM and healthy individuals and thus provide a potential approach to identify people with unrecognized oHCM.

## Results

### Patient characteristics

Out of 21 patients in PIONEER-HCM, two were excluded from the digital substudy because of sensor errors during data collection. The 19 enrolled oHCM patients were 22–70 years old, and nine (47%) were women (Table 1). Participants had left ventricular hypertrophy (interventricular septal thickness 1.64 ± 0.20 cm) with severe resting left ventricular outflow tract (LVOT) obstruction (peak pressure gradient 70.1 ± 42.8 mmHg). All were in sinus rhythm at the time of sensor recording. The 64 healthy volunteers enrolled in MYK-491-001 comprised the control group. They were 18–49 years old (38% women), and none had evidence of left ventricular hypertrophy (interventricular septal thickness 0.83 ± 0.13 cm), LVOT obstruction or other cardiovascular disease.

**Table 1:** Baseline characteristics

	oHCM patients	Healthy volunteers
Number enrolled	19	64
Age, mean (SD), years	57.5 (14.3)	28 (7.4)
Sex, # female (%)	9 (47)	24 (38)
Heart rate, mean (SD), bpm	72 (11)	59 (9)
Resting LVEF, mean (SD), %	73 (6.2)	63 (4.1)
Septal thickness, mean (SD), cm	1.64 (0.20)	0.83 (0.13)
Resting LVOT gradient, mean (SD), mmHg	70.1 (42.8)	NA

### Analysis of PPG waveforms

**Fig. 1:**
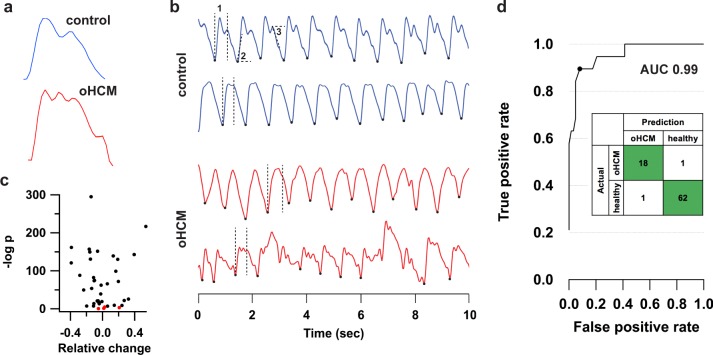
Differences in photoplethysmography tracings between oHCM patients and healthy volunteers. **a** Single beats extracted from the PPG recordings and **b** 10 s continuous recordings illustrating differences in waveform morphology between two representative healthy subjects and two oHCM patients. Markers indicate separation between beats detected by an automated algorithm. Example morphometric features are shown as follows: (1) systolic ejection time, (2) slope of systolic rise, and (3) slope of diastolic decline. **c** Plot of the magnitude and statistical significance of the difference in feature values between healthy controls and oHCM patients for 42 analyzed pulse features. The 38 features with Bonferroni-corrected *p* < 0.05 are colored black and the remaining four are shown in red. **d** Receiver-operator curve with marker indicating the cutoff point used to derive the embedded confusion matrix

Continuous PPG recordings revealed differences in pulse wave patterns between control subjects and oHCM patients. In individual beats (Fig. 1a), pulse wave traces from oHCM patients often had a steeper initial rate of rise and contained multiple peaks of variable intensity. When patterns were examined across multiple beats, pulse wave traces from oHCM patients showed more frequent irregularly shaped beats and greater variability from beat to beat, including with respiration, than those from healthy controls (Fig. 1b). A set of 42 morphometric features was algorithmically extracted from all tracings (Fig. 1b) of which 38 differed significantly between groups of healthy volunteers and oHCM patients (Fig. 1c), including measures of systolic ejection time, slope of the systolic upstroke, and respiratory variation. These data suggest that, in aggregate, beats from oHCM patients are morphologically distinct from those of healthy volunteers.

### Development of machine learning classifier to detect oHCM

We proceeded to develop an automated classifier that could distinguish between recordings from oHCM patients and healthy volunteers. Although significant differences were found in many morphometric pulse features, the substantial beat-to-beat variability within individual traces (as illustrated in Fig. 1b) limited the performance of classifiers based on averaging beats across a recording. To best account for this heterogeneity, we used a multi-instance classifier to calculate an “oHCM score” for each recording (see Methods). After training and cross-validation, the model achieved a C-statistic for oHCM detection of 0.99 (95% CI: 0.99–1.0). At an operating threshold that optimizes the sum of sensitivity (95%) and specificity (98%), the model correctly classified 18/19 patients with oHCM and 63/64 healthy volunteers (98% accuracy) (Fig. 1d). The final model thus achieved discrimination between patients with oHCM and healthy controls.

## Discussion

In this study, we developed an automated machine learning classifier to detect oHCM in PPG signals collected from a wrist-worn optical biosensor in patients who had resting outflow tract obstruction. This work builds on decades of investigation into oHCM hemodynamics and integrates them with contemporary advances in biosensor technology and machine learning to create a noninvasive strategy to detect disease outside of a clinical setting. Indeed, the algorithm combines features corresponding to known hemodynamic abnormalities in HCM (e.g., rate of systolic pressure rise and systolic ejection time) with morphological features extracted from the PPG signal.

This proof-of-principle study provides motivation for more comprehensive trials to better characterize the PPG signature of oHCM and to explore other structural heart diseases. Strengths of this study include its conduct at multiple sites by investigators who were centrally trained to obtain high-quality PPG recordings in a consistent manner, and the synchronization of PPG with echocardiograms that were performed by centrally trained sonographers and interpreted in a core laboratory. However, the current study is limited by its small size and the fact that data were collected from two separate studies. There were differences in age, gender, and beta blocker utilization between cohorts, although none were correlated with oHCM score in subgroup analyses. Furthermore, the changes observed in oHCM are not similar to previously described age-dependent changes in PPG waveforms.^11^ Future studies incorporating larger cohorts are required to study the impact of age and gender in oHCM PPG signals more extensively. It also cannot be excluded that other differences between the two studies could have potentially affected the results of this analysis. Future studies comparing demographically matched cohorts evaluated under the same conditions will help resolve this uncertainty. Finally, the Leave-One-Group-Out cross-validation method may overestimate performance of the classifier, so further validation studies incorporating strictly separated training and testing datasets will be required to confirm the initial results presented in this paper.

The increasing ubiquity of wearable devices with PPG sensors increases the feasibility and potential impact of implementing an algorithm to detect oHCM from PPG signals. The high rate of undiagnosed oHCM, combined with the high cost of global screening using current technology, underscore the need for broadly available, inexpensive screening methods for this disease. More effective identification of seemingly healthy individuals with oHCM who may be at risk of developing cardiac morbidity would be of significant benefit.

## Methods

### Study design

The PIONEER-HCM digital substudy was conducted at five HCM referral centers in the US as part of a phase 2 clinical trial to evaluate the effects of treatment with mavacamten, a small molecule myosin inhibitor, in patients with symptomatic oHCM (NCT02842242).^12^ The study enrolled 21 patients ages 18–70 with HCM, NYHA class II–III symptoms, and a resting LVOT gradient greater than 30 mmHg. Patients in PIONEER cohort A (*n* = 11) were on no concomitant cardiac medications; in cohort B (*n* = 10), background beta blockers were permitted. Control data were obtained from all healthy volunteers (*n* = 64) enrolled in the MYK-491-001 study to evaluate MYK-491, a small molecule myosin activator (NCT03062956). These participants were identified as free of cardiovascular disease by history, physical examination, ECG, and echocardiography. Both trial protocols were reviewed and approved by the relevant ethics committees. An independent data monitoring committee regularly reviewed the study data to help identify emerging safety or conduct issues. All patients provided informed consent, and the studies were conducted in accordance with the provisions of the Declaration of Helsinki and the International Conference on Harmonization Good Clinical Practice guidelines.

### Data collection and analysis

Study subjects underwent resting echocardiography with standard two-dimensional, M-mode, and Doppler imaging by trained sonographers. Studies were read by a central laboratory (Brigham and Women’s Hospital, Boston, MA).

PPG signals were collected for 5 min (1–5 recordings per participant) at rest using an investigational wrist-worn biosensor (Wavelet Health, Mountain View, CA) at either the screening visit or on Day 1 of the study prior to receiving investigational drug.^13^ PPG signals from all patients were acquired by a single investigator at each site, who underwent centralized training on a documented procedure that minimizes the impact of differences in environmental factors including ambient light and temperature. All devices ran identical firmware and signal processing methods to obtain high-quality signals. Signals were transmitted by Bluetooth to an iPad and uploaded to a cloud database for analysis.

Recordings were segmented into beats using an automated algorithm, and a multi-instance classifier was trained to assign each recording an oHCM score based on qualified beats (instances).^14^ Briefly, a set of 42 morphometric pulse features was extracted into a feature vector for each beat. The multiple-instance learning via embedded instance selection (MILES) method^15^ was used. It consists of (i) transforming feature vectors from all beats in a recording into a single vector per recording and (ii) fitting the resulting vectors with a support vector machine. For evaluation of the final MILES model, we employed Leave-One-Group-Out cross-validation with nested hyperparameter tuning,^16^ which in turn used 68-fold cross-validation with random selection of training and testing cohorts (70% testing/30% training). In summary, for each patient in the dataset, the model was trained and tuned using all recordings except for that patient’s.^17^ The accuracy, sensitivity, specificity and area under the curve of this model were evaluated.

### Reporting summary

Further information on research design is available in the Nature Research Reporting Summary linked to this article.

## Supplementary information

Reporting Summary

## Data Availability

No data repository is available for this digital substudy. Requests for the complete deidentified patient dataset and clinical protocols addressed to the Corresponding Author would require evaluation on an individual basis. The authors made the appropriate materials available to the editorial staff during the review process for verification of results.

## Supplementary information

**Supplementary information** accompanies the paper on the *npj Digital Medicine* website (10.1038/s41746-019-0130-0).

## Code availability

Restrictions apply regarding the use of this code. It is available from Dr. Dur upon reasonable request.
